# The Role of Airway and Endobronchial Ultrasound in Perioperative Medicine 

**DOI:** 10.1155/2015/754626

**Published:** 2015-12-14

**Authors:** Jiri Votruba, Petra Zemanová, Lukas Lambert, Michaela Michalkova Vesela

**Affiliations:** ^1^First Department of Tuberculosis and Respiratory Care, 1st Medical Faculty and General University Hospital, U Nemocnice 2, 128 08 Prague, Czech Republic; ^2^Department of Radiology, 1st Medical Faculty and General University Hospital, U Nemocnice 2, 128 08 Prague, Czech Republic; ^3^Department of Anesthesia and Intensive Medicine, Na Bulovce Hospital, Budínova 2, 180 00 Prague, Czech Republic

## Abstract

Recent years have witnessed an increased use of ultrasound in evaluation of the airway and the lower parts of the respiratory system. Ultrasound examination is fast and reliable and can be performed at the bedside and does not carry the risk of exposure to ionizing radiation. Apart from use in diagnostics it may also provide safe guidance for invasive and semi-invasive procedures. Ultrasound examination of the oral cavity structures, epiglottis, vocal cords, and subglottic space may help in the prediction of difficult intubation. Preoperative ultrasound may diagnose vocal cord palsy or deviation or stenosis of the trachea. Ultrasonography can also be used for confirmation of endotracheal tube, double-lumen tube, or laryngeal mask placement. This can be achieved by direct examination of the tube inside the trachea or by indirect methods evaluating lung movements. Postoperative airway ultrasound may reveal laryngeal pathology or subglottic oedema. Conventional ultrasound is a reliable real-time navigational tool for emergency cricothyrotomy or percutaneous dilational tracheostomy. Endobronchial ultrasound is a combination of bronchoscopy and ultrasonography and is used for preoperative examination of lung cancer and solitary pulmonary nodules. The method is also useful for real-time navigated biopsies of such pathological structures.

## 1. Introduction

Ultrasound, as a noninvasive radiological assessment, was first used in 1953 when two Swedish cardiologists performed the first successful ultrasonographic examination of the heart [1]. With the development of technology, ultrasound has been established as a rapid bedside method in preoperative assessment and perioperative practice and also in the intensive care setting.

Two modalities of respiratory system ultrasound are currently used for preoperative assessment, for postoperative examination, and for real-time guidance in some interventional airway procedures. Transcutaneous ultrasound includes translaryngeal and transtracheal ultrasound examinations which have been used for conventional scans of the oral cavity, vocal cords or trachea [2], and transcutaneous ultrasound assessment of the lungs. Endobronchial ultrasound is a novel tool combining bronchoscopic evaluation with tissue ultrasonography and has been used mainly for preoperative diagnostic purposes [3].

The following paragraphs will focus on the detailed applications of both conventional and endobronchial ultrasound in perioperative practice.

## 2. Methodology

The authors performed an extensive literature search using the PubMED and SCOPUS databases. The following terms were used as search parameters: “ultrasound” AND “airway”, “respiratory”, “vocal cords”, “trachea”, and “endobronchial”. The selected language was English. In total, 475 articles were retrieved and, after careful selection, 83 of them were studied in detail for this review. Mainly levels I and II evidence articles and systematic reviews, if available, were included.

## 3. Examination with Conventional Ultrasound Techniques

Conventional external ultrasound techniques are important tools for the preoperative assessment of the airway [4] and for evaluation of endotracheal tube positioning [5]. They may also be useful for real-time guidance during interventional airway procedures, such as cricothyroidotomy or percutaneous dilatational tracheostomy [6]. In the postoperative period, ultrasound may help in the diagnosis of airway obstruction or pathological changes of the vocal cords [7].

### 3.1. Choice of Probe and Visualization

Choice of the appropriate ultrasound transducer and suitable mode on the ultrasound machine is basic prerequisite for successful visualization of the airway structures. A linear, high-frequency (5–14 MHz) transducer is used for visualization of the superficial anatomical structures, such as the cricoid cartilage, cricothyroid membrane, tracheal rings, or trachea itself [8].

Different transducers are used for the location of more deeply located anatomical structures, such as the base of the tongue, epiglottis, vocal cords, or arytenoid cartilages. Microconvex ultrasound probes with a working frequency of 4–10 MHz or convex (curvilinear) probes with a frequency of between 3 and 8 MHz are most frequently used in this case [9].

### 3.2. Airway Assessment with Ultrasound

Preoperative airway assessment has traditionally been carried out using various tests, such as Mallampati classification, Wilson grading, evaluation of neck movements, and measurement of thyromental or hyomental distance. None of these tests have been found to be sufficiently specific or sensitive, and often the combination of two or more tests is used in order to obtain more precise information [10]. Magnetic resonance imaging (MRI) of the airway may be used for obtaining sophisticated information in those patients with predicted difficulty. However, MRI is not a dynamic method; it is not available immediately and not feasible for every patient. Ultrasound may offer some advantages in preoperative airway evaluation. It is a bedside method, relatively easy, cheap, and reasonably fast and it can also provide real-time and dynamic information about the airway anatomy throughout the breathing cycle. Any organs or cavities filled with the air are not penetrated by an ultrasound beam, therefore all structures visible beyond the interface of tissue and air are considered as artifacts. Many anatomical structures of the oral cavity, oropharynx, larynx, or subglottic area may be well visualized using ultrasound. However, those structures located posterior to the tissue-air interface such as the dorsal part of pharynx, posterior commissure, or the posterior wall of the trachea cannot be evaluated with the ultrasound [4]. Ultrasonographic evaluation of the airway may be divided into several sections.

#### 3.2.1. Examination of the Oral Cavity and Pharynx

**Figure 1:**
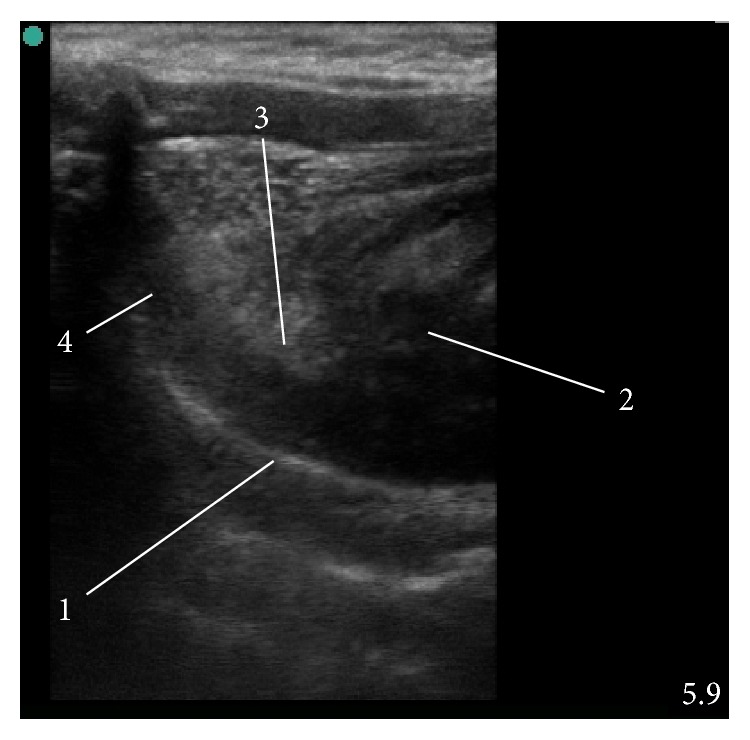
Ultrasound visualization of the tongue and floor of the mouth. (1) Hard palate, (2) tongue, (3) epiglottis, and (4) laryngeal inlet. The picture is “upside down” because the examination is performed via the sublingual window and therefore the hard palate is the deepest structure on the image.

Various structures of the oral cavity and hypopharynx may be visualized using a submental window. Curvilinear (convex) or microconvex probes are usually used. The hyoid bone lies quite superficially and can be visualized using a transversal midline position as a linear structure (inverted U-shape) or by using a parasagittal approach [4]. The epiglottis is best visualized through the thyrohyoid window either with a median transverse or with parasagittal approach using a linear probe. The median transverse approach offers better visualization of the structure [11]. Alternatively, the epiglottis is also visible with a curvilinear probe and by using a sagittal projection between the hyoid bone and the tip of the mandible. The tongue and its adjacent structures play a very important role in difficult airway management. Tongue enlargement or lingual tonsillar hyperplasia may cause significant problems with direct laryngoscopy. The tongue and surrounding structures are best viewed using submental transversal or parasagittal projections. The tongue is located deep to the mylohyoid, geniohyoid, hyoglossus, and genioglossus muscles (Figure 1) [11].

#### 3.2.2. Examination of the Vocal Cords

**Figure 2:**
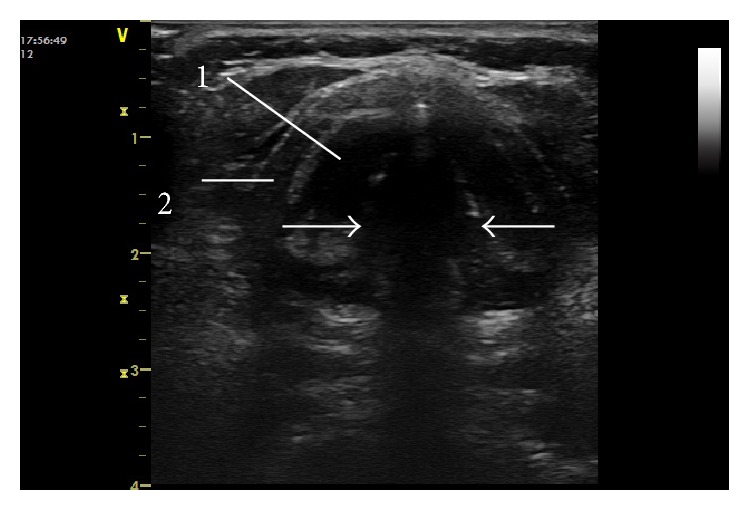
Ultrasound imaging of the relaxed vocal cords (white line with arrows). (1) Vocal muscle. (2) Thyroid cartilage.

The vocal cords can be visualized using the linear or microconvex probe. The transducer is placed in the midline at the level of cricoid cartilage and the vocal cords are identified as inverted, “V” shaped echogenic structures (Figure 2).

The vocal cords are usually assessed in two stages—initially during shallow breathing, which allows evaluation of their shape, and, for the presence of edema, potential glottic mass, nodule, or polyp. The second stage involves evaluation of the movement during phonation [12]. Other structures adjacent to the vocal cords such as the aryepiglottic folds, anterior commissure, vocal process of arytenoid cartilages, or ventricular folds may be located using this transthyroid window. A standard B-mode is routinely used for the assessment of vocal cords while combination of the B-mode with Doppler imaging may help to evaluate function of the cords [7]. A novel mode using ultrasound, Nakagami imaging, is a functional method for visualization of the relative concentrations of collagen and elastic fibres and has been developed for better evaluation of the biomechanical properties of the vocal cords [13].

#### 3.2.3. Cricothyroid Membrane and Trachea

**Figure 3:**
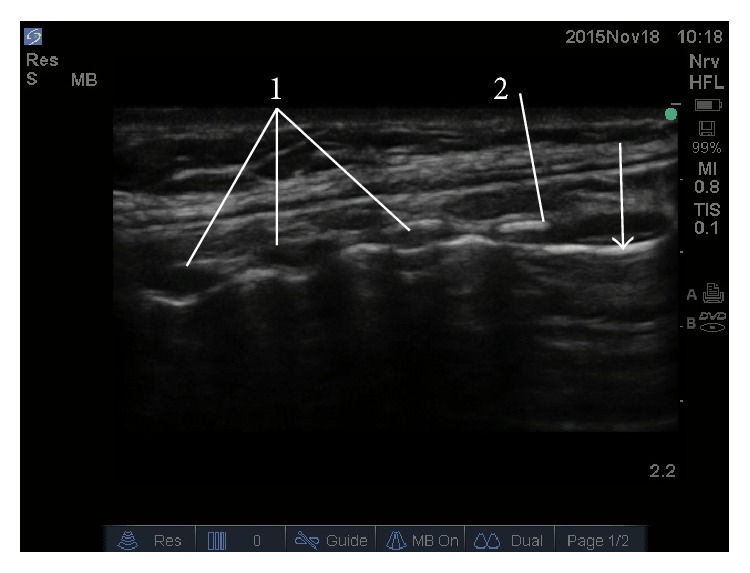
Cricothyroid membrane (white arrow) is best visualized using median or paramedian sagittal view. (1) Tracheal ring. (2) Cricoid cartilage.

The diameter of the subglottic space, where stenoses may often be located, can be assessed using a linear probe [14] using a midline transverse view. This measurement can serve as a guide for selection of the correct size of tracheal tube because the subglottic space is the narrowest part of trachea. The cricothyroid membrane is best located using a median or paramedian sagittal view [4]. This membrane is usually located very superficially and a high-frequency linear probe is suitable for this examination. The hyperechogenic membrane lies between two bony hypoechogenic structures—the cranially located lower border of the thyroid cartilage and caudally located cricoid cartilage (Figure 3).

**Figure 4:**
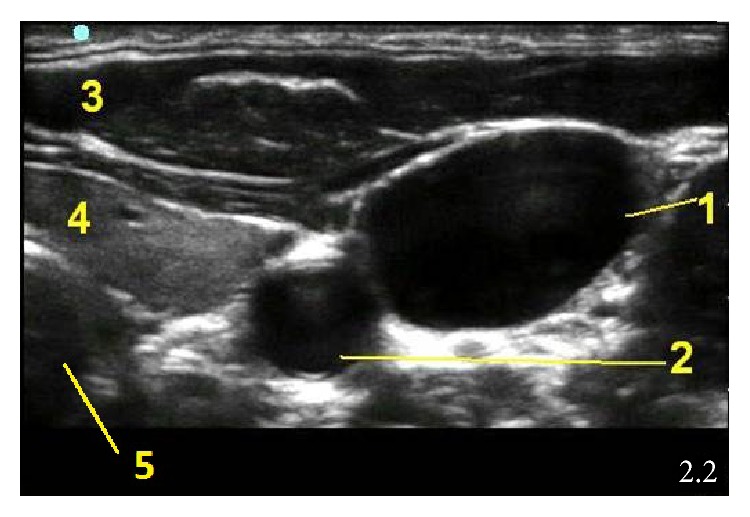
Transverse neck ultrasound image at the level of C6 vertebra. (1) Internal jugular vein, (2) common carotid artery, (3) subcutaneous tissue, (4) thyroid gland, and (5) trachea.

The anterior parts of the tracheal rings are seen using the same views with slight movement of the probe caudally. They have the appearance of small oval structures located close to each other. Any masses located below the cricothyroid membrane or inside of the anterior part of trachea may be located using ultrasound. The transverse view at the level of the C6 vertebra provides very good information about the topographic anatomy of the trachea and surrounding structures. In addition to the trachea, performing physician can also visualize the esophagus, thyroid gland, common carotid artery, internal jugular vein, and cervical muscles (Figure 4).

### 3.3. Clinical Applications of Airway Ultrasound

Ultrasound of the airway is not as frequently used as ultrasound for other perioperative indications, such as regional anesthesia, establishment of vascular access, or evaluation of cardiac performance. However, its availability and portability and the increasing experience of anesthesiologists with ultrasound in general have led to its growing utilization in this field.

#### 3.3.1. Prediction of Difficult Laryngoscopy

A number of articles, with sometimes contradictory results, have focused on prediction of difficult laryngoscopy using ultrasound. A measurement of pretracheal tissue mass has been found to be an important factor in difficult laryngoscopy prediction in morbidly obese patients with BMI over 35 kg·m^−2^ [15]. Patients with difficult laryngoscopic views had a mean pretracheal thickness of 28 mm in comparison with 17.5 mm in those who presented with easy laryngoscopy. Only the pretracheal tissue mass and increased neck circumference correlated with difficult laryngoscopic views. However, another study performed in an obese population failed to confirm any correlation between the measurement of pretracheal soft tissue mass with the ultrasound and intraoperative difficult laryngoscopy [16]. Measurements of anterior neck soft tissue mass at the levels of hyoid bone, thyrohyoid ligament, and anterior commissure were found to be independent predictors of difficult laryngoscopy in 203 patients with a mean BMI within normal values [17]. Another study found a positive correlation between easy laryngoscopy (grades 1 and 2) and visibility of the hyoid bone using sublingual ultrasound [18]. Wojtczak used ultrasound for preoperative assessment of the hyomental distance and tongue volume in a pilot study and found that patients with difficult laryngoscopy had significantly shorter hyomental distance but no differences in tongue volume [19].

#### 3.3.2. Vocal Cord Paralysis and Other Pathologies

Preoperative and postoperative assessment of mobility of the vocal cords are an important tool for airway management. Vocal cord movements may be visualized using rigid or flexible laryngoscopy or, noninvasively, by using ultrasound. Miles first described the use of translaryngeal ultrasound for assessment of vocal cord movements in 1989 [20].

**Figure 5:**
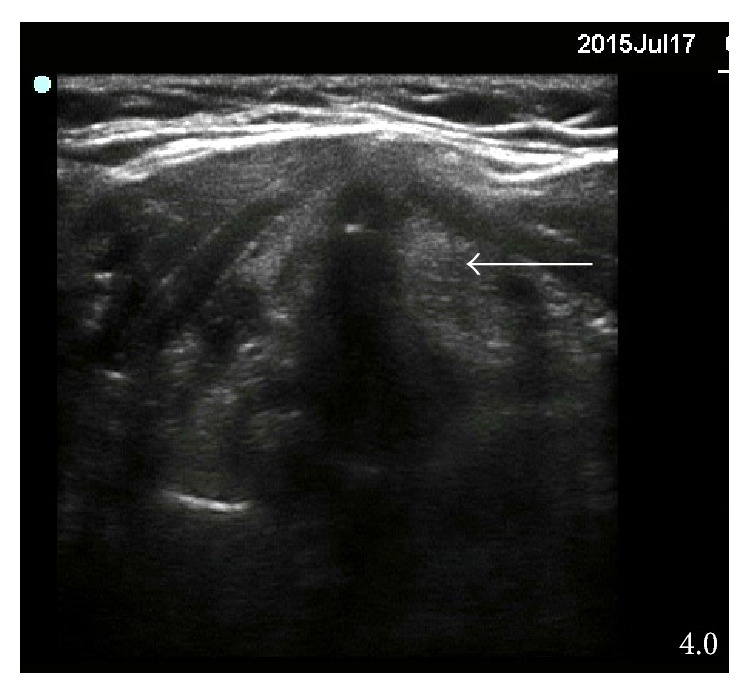
Unilateral vocal cord palsy (white arrow).

Translaryngeal assessment of vocal cord movements is a useful and reliable method after thyroid and parathyroid surgeries. This method may be a useful tool in diagnosing superior laryngeal nerve or recurrent laryngeal nerve injuries (Figure 5) [21].

However, success rate in visualization of true vocal cords is significantly lower than that of the false vocal cords or arytenoids [22]. Postoperative translaryngeal ultrasound has been shown to diagnose vocal cord palsy in 94% of female patients but only in 53% of males [23]. The success rate of examination is also significantly lower in patients with calcification of the thyroid cartilage [24]. Wong et al. found a high correlation between asymmetry of the vocal cords on ultrasound views and postoperative voice changes [22]. Another application of translaryngeal ultrasound in perioperative medicine is prediction of postextubation stridor [25] by measurement of the width of the laryngeal air column [26]. Cheng et al. recommend preoperative ultrasound of the vocal cords for a selection of patients with abnormal findings, such as asymmetry of the cords or their swelling before subsequent microlaryngoscopy [27]. 3D ultrasound can further help to improve efficacy in diagnosing of postoperative vocal cord paresis [28].

Rubin et al. reported that translaryngeal ultrasound showed high accuracy in patients without any pathology of the vocal cords but failed to diagnose unilateral vocal cord lesion in less than 50% of patients [29]. A significantly higher success rate in diagnosing benign lesions of the vocal cords—87%—was reported with more advanced US technologies [30]. The first paper recommending translaryngeal ultrasound for evaluation of normal anatomy of the vocal cords and various pathological findings in children and infants was published in 1991 [31]. Spadola Bisetti et al. reported a high efficacy in finding benign vocal cord lesions such as cysts, polyps, and papillomatosis in pediatric patients with translaryngeal ultrasound [32]. 2D and 3D ultrasound examinations may be used for assessment of the fetal larynx and pharynx with the aim of diagnosing upper airway pathologies [33].

#### 3.3.3. Subglottic Space Measurement and Tracheal Tube Selection

The first animal experiments on measurement of the subglottic space with B-mode ultrasound were published in 2000 [34]. Subsequent studies have evaluated the accuracy of ultrasound measurement of this space in children [35] and adults [14]. Lakhal et al.'s study [14] also demonstrated a very good correlation between ultrasound and magnetic resonance imaging (MRI) measurements. These studies concluded that the method may reliably determine the size of endotracheal tube in adults but it underestimates the size in pediatric patients. Newer studies on children confirm the fact that ultrasound measurement of the upper subglottic diameter correlates highly with the desired outer diameter of both uncuffed and cuffed endotracheal tubes [36, 37]. US-measured minimal subglottic diameter showed very good correlation with the appropriate endotracheal tube outer diameter in children older than 12 months but a worse correlation in younger pediatric patients [38]. Bae et al. concluded that, even with the ultrasound, the success rate of a correct choice of endotracheal tube in small children does not exceed 60% [39].

Modern applications of ultrasound such as the three-dimensional (3D) reconstructions allow better assessment of airway anatomy including the anterioposterior (AP) diameter which is not possible to visualize using conventional ultrasound. All measurements seem to correlate very well with the MRI scanning [40].

**Figure 6:**
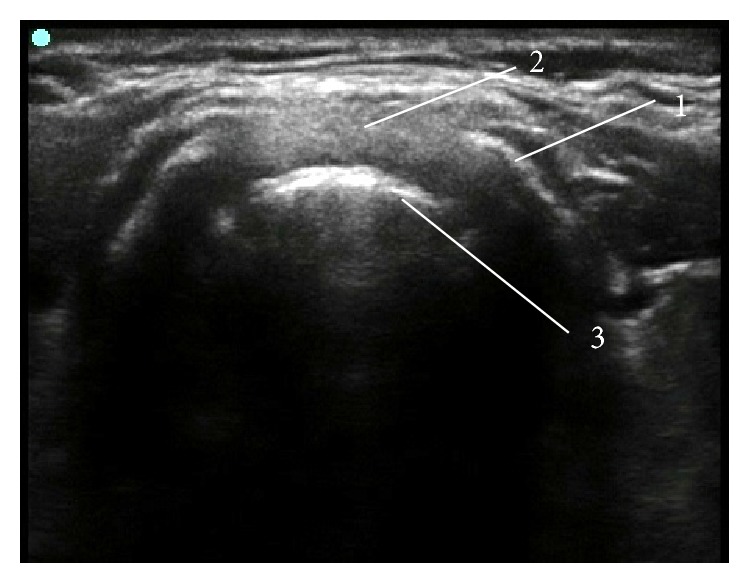
Ultrasound visualization of a stent inserted into the tracheal stenosis. (1) Tracheal wall, (2) intratracheal stenotic tissue, and (3) anterior part of the tracheal stent.

Ultrasound may also be a very useful tool in evaluation of the severity and extent of tracheal stenosis (Figure 6).

This was initially documented in a case of a woman presenting at the emergency department with progressive shortness of breath [41]. A bedside ultrasound was able to locate the stenosis and estimate the narrowest diameter of the trachea. In a study on 26 patients, conventional B-mode ultrasound has shown 88.5% correlation with fibreoptic findings and a 80.7% correlation with computed tomography (CT) [42].

#### 3.3.4. Confirmation of Endotracheal Tube Position

Ultrasound may be reliably used for direct or indirect confirmation of the correct placement of an endotracheal tube. One of the indirect methods is based on confirmation of the incorrect position of the tube in the esophagus. The specificity of ultrasound detection of the tube inside the esophagus is extremely high [5]. Ultrasound confirmation of tracheal placement of the tube may be performed using real-time ultrasound with the probe placed over the anterior part of the neck or after intubation with the aim being to find the shadow caused by the endotracheal tube inside the trachea. In the operating room, scanning of the neck at the level of the suprasternal notch detected both tracheal intubation and esophageal tube placement with very high specificity and sensitivity [43]. Abbasi et al. found a very high specificity and sensitivity with both dynamic (real-time) and static ultrasonography in confirmation of tracheal tube position in the emergency department [44]. Transcricoid US examination was used for dynamic evaluation while a suprasternal notch view was employed for postprocedural static evaluation. Transtracheal sonography showed 98.1% correlation with confirmation of emergency endotracheal tube placement [45]. Chou et al. recommends T.R.U.E. (tracheal rapid ultrasound exam) as a reliable and fast method for confirmation of correct and incorrect tracheal intubation [46]. This method can also be used without any difficulties during cardiopulmonary resuscitation [47]. Muslu et al. reported 100% sensitivity and specificity for tracheal and esophageal intubation in the operating room [48]. Another indirect method is based on looking for the signs of ventilation at the level of the pleurae [49] or diaphragm muscle [50]. Lung sliding/lung pulse is caused by movement of the lung in relation to the chest wall. Presence of this sign on the ultrasound is an indirect sign of correct tracheal tube placement [49]. Park et al. recommend a combination of transtracheal ultrasound for confirmation of the tube location in the trachea and transthoracic sonography for evaluation of lung sliding [51]. Transthoracic ultrasound has been successfully used in confirmation of correct double-lumen tube in thoracic surgical procedures [52]. In pediatric practice, translaryngeal ultrasonography can help to confirm correct laryngeal mask airway placement or its malposition [53].

#### 3.3.5. Location of Cricothyroid Membrane and Cricothyrotomy

Emergency cricothyrotomy is a life-saving procedure in acute upper airway obstructions or in “cannot intubate, cannot ventilate” situations [54]. However, correct location of cricothyroid membrane can prove difficult in some patients. In one paper, use of three different landmark or palpation methods showed only 46–62% success rate [55]. Preemptive ultrasound location of the cricothyroid membrane in the patients with expected difficulty to manage airway is easy and reliable technique [56, 57]. You-Ten et al. compared the palpation method with ultrasound location of the membrane in obese peripartum females and showed only 39% accuracy for palpation versus 100% accuracy with the use of ultrasonography [56]. Campbell et al. reported a more difficult location using palpation of the cricothyroid membrane in females whereas the ultrasound technique showed similarly high efficacy in both genders [57]. Another study also reported the lowest success rate of the cricothyroid ligament palpation in obese women [58].

A possibility of real-time ultrasound guidance for the puncture of cricothyroid membrane and bougie-assisted cricothyroidotomy (BACT) was first studied on cadaver models [59]. The participants were able to locate the membrane within 5 sec and the mean time for completion of the procedure was 26 sec. Suzuki et al. used real-time ultrasound for percutaneous insertion of a cannula through the cricothyroid membrane in the acute setting [60].

Ultrasound may be used for the location of cricothyroid membrane in emergency departments. The method shows high accuracy and its learning curve is very shallow [61].

#### 3.3.6. Percutaneous Dilatational Tracheotomy

Percutaneous dilatational tracheotomy (PDTS) is one of most commonly performed procedures in intensive care units. Ultrasound imaging may be used before the procedure for identification of the trachea and its adjacent anatomical structures [62]. It has been used for preprocedural assessment since 1999 to locate the trachea, isthmus of the thyroid gland, and any vascular structures in the anterior neck area [63]. Ultrasound may also help to find the most appropriate level for tracheotomy with determination of a safe trajectory for incision and preparation [64]. A change of the puncture site determined with a landmark technique was recommended in 24% of patients when ultrasound was used prior to puncture in the ICU [65]. Protocols have already been established in some institutions for the strict application of US scanning before PDTS [66].

The feasibility of ultrasound-guided puncture of the trachea in real-time was first studied using fresh cadavers [67]. The neck was scanned with a linear probe giving a longitudinal visualization of the trachea. Dinh et al. compared US-guided approach with a landmark technique and concluded that the use of ultrasound increased the percentage of midline punctures, decreased the total number of attempts, and aided in placing the tracheotomy tube safely into the trachea [68]. These findings were confirmed by another study that also showed a lower number of complications in the US-guided group [69]. US-guided PDTS have been also compared with bronchoscopy-guided PDTS in a retrospective cohort trial with similar results apart from shorter procedure time in the ultrasound group [70]. The influence of the ultrasound use on the complication rate in the PDTS was studied by Rajajee and colleagues [71]. The authors showed a significantly lower incidence of potential complications, such as bleeding, early tube dislocation, or granuloma in the US-guided group. Despite these positive results, a recent review concluded that US-guided PDTS is a promising method but its superiority over other techniques should be confirmed in robust randomized trials [6].

## 4. Endobronchial Ultrasound

The last decade has seen rapid development of novel ultrasonography technologies. These methods have found roles in the preoperative assessment of cancerous and noncancerous lesions in various locations of the body. Pulmonary pathologies such as tumors and affected lymph nodes were mainly diagnosed in the past using CT and positron emission tomography/computed tomography (PET/CT). However, CT had not been successful in evaluation of nodal staging and airway wall infiltration [72]. PET/CT combines anatomical information from CT and metabolic information obtained from PET scanning and therefore it is potentially an accurate and noninvasive method in the staging of cancer. This method also may improve the mediastinal lymph node staging. However, both specificity and sensitivity are far from being high enough to abandon biopsy confirmation of disease.

The following factors are challenging in the preoperative assessment of any respiratory tract lesion. First of them is a precise, fast, safe, and affordable tumor and nodal staging. The second factor is proving or excluding tumor infiltration of the airway wall and the third one is the diagnosis of solitary pulmonary nodules (SPNs). This is an isolated lung abnormity on the chest X-ray, smaller than 3 cm, surrounded by normal lung tissue, and not associated with any other pulmonary pathology.

These are reasons why new imaging tools for preoperative thoracic cancer staging were sought in the early 1990s. Existing bronchoscopic techniques for nodal staging—TBNA (transbronchial needle aspiration) and TBB (transbronchial biopsy)—did not have sufficient efficacy in obtaining diagnostic tissue. Transbronchial fine needle aspiration for nodal staging was found to have a sensitivity of 57% for lymph nodes greater than 10 mm with a specificity of 99% [73]. In a meta-analysis performed by Holty et al., the pooled sensitivity for TBNA mediastinal staging was even lower, at 39% (95% CI, 17–61%), with a pooled specificity of 99% [74]. Electromagnetic navigation bronchoscopy (ENB) allows for guidance of the bronchoscope to the solitary pulmonary lesion through a reconstructed CT image. Overall sensitivity of this technique was shown to be 71% [75]. The ENB system is still very expensive and so its use is naturally restricted.

In early 1980s, the first mechanical radial instruments for endoscopic ultrasound were introduced in gastroenterology [76]. The first dedicated endobronchial ultrasound system was made commercially available in 1999. It consisted of a balloon catheter with a rotating ultrasonic head. As with any other ultrasound device it consists of a system of piezoelectric crystals—transducer and a processor. The current widespread use of endobronchial ultrasound (EBUS) is due to an easier-to-interpret and more user-friendly device which was developed in Japan in 2005. This linear EBUS has the ability to facilitate real-time transbronchial needle aspiration under direct ultrasound guidance through the working channel of the endoscope [77]. However, during the last 5 years we have witnessed continuous return of slightly remastered radial EBUS miniprobes to clinical practice.

All interventional bronchoscopies and an increasing number of diagnostic bronchoscopies are currently performed under general anesthesia for the comfort of the patient and the convenience of the operator. Supraglottic airway devices such as laryngeal masks [78] or i-gel [79] may be used instead of rigid bronchoscope insertion.

### 4.1. Radial EBUS—in the Search of SPNs

With the above-mentioned increase in frequency of radiologically diagnosed SPNs, physicians are creating an increased demand for tissue diagnosis of indeterminate nodules. It is important to be both accurate and efficient in the preoperative diagnostic evaluation of SPN because rapid resection of malignant tumor can be life-saving. In patients with resected malignant nodules, the 5-year survival rate may be as high as 80%. With the widespread use of fluoroscopic guidance for TBB, endoscopists need a device for real-time point monitoring, a device which would give clear positive or negative information. Main issues with the technique are if the catheter is correctly located in the node and if the biopsy should be taken from this area.

**Figure 7:**
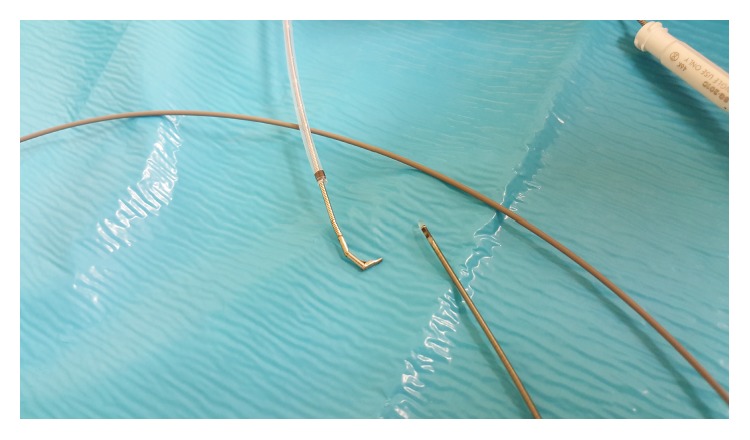
A radial EBUS miniprobe.

EBUS miniprobes are currently offered with outer diameters of 1.4 mm and 1.7 mm and are therefore available for the use in the periphery of the lung (Figure 7).

These are introduced through the working channel of a bronchoscope and offer a 360 degrees' view. A 20 MHz working frequency is commonly used in lung tissue. The probe can be reused up to 80 times if used with care. The navigation is a rather tricky procedure consisting of introducing the steerable guiding device through the Teflon catheter which is then inserted through the working channel of the endoscope. After ideal positioning of the catheter in SPN according to fluoroscopy, the guiding device is replaced by a radial EBUS probe and its positioning inside the nodule is confirmed. Subsequent biopsy is carried out with forceps or using a cryotechnique. This is a complicated and inaccurate method and as such it can be quite time consuming.

According to the literature data, radial EBUS dramatically improves the success rate of fluoroscopically navigated transbronchial procedures for diagnosing SPNs [80]. In a randomized trial which compared routine bronchoscopic transbronchial biopsy under fluoroscopy with radial probe EBUS facilitated TBB, the yields were 52% and 76%, respectively [81]. From a therapeutic perspective, navigational bronchoscopy has been utilized to place fiducial markers in order to carry on stereotactic body radiation therapy (SBRT) [82].

Nowadays, EBUS is perceived as irreplaceable tool by most bronchoscopists. The principal reason for this is the ability of EBUS to help physicians in many difficult situations.

### 4.2. Accessing the Extent of Airway Invasion

EBUS has enabled the bronchoscopist to extend his vision beyond the tracheobronchial wall. As stated before, CT is usually unable to predict the extent of pathologic tumor invasion into the airway wall. For many therapeutic purposes it is crucial to know if the tumor is extending beyond the cartilaginous layer or if it remains within the submucosal layers. This finding is crucial for differentiation between early and invasive lung cancer. The extent of tumor invasion can provide important information about the resectability of a lesion. EBUS has a specificity of 100%, a sensitivity of 89%, and an accuracy of 94% in differentiating external compression of the airway from tumor infiltration, in comparison to CT, which has a specificity of 28%, a sensitivity of 75%, and an accuracy of 51% [77].

### 4.3. Diagnosis of Mediastinal and Hilar Lesions

**Figure 8:**
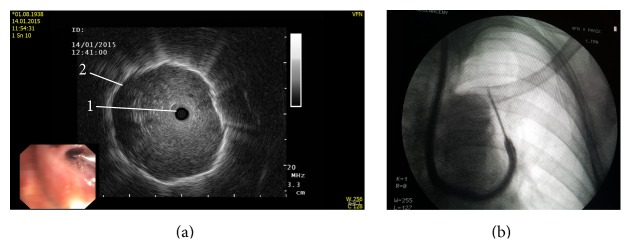
Endobronchial ultrasound-navigated lung biopsy. (a) Bronchoscopic and ultrasound image, (1) bronchus, and (2) external border of the tumor. (b) X-ray control of the procedure.

Linear probe EBUS (Figure 8) is an efficient device allowing real-time ultrasonography guidance during needle insertion into a suspected nodal area.

Preoperative EBUS-TBNA biopsies can be obtained from all following lymph node locations—2L, 2R, 3, 4R, 4L, 7, 10R, 11R, and 11L. 2013 ACCP guidelines for the diagnosis and management of lung cancer [83] clearly state that EBUS-TBNA should be used in primary staging of lung cancer whenever possible. However, a low negative predictive value still necessitates surgical staging before deciding on thoracotomy.

## 5. Conclusion

More prospective randomized studies are needed to confirm the position of conventional ultrasound in prediction of difficult airway management. Future research should be focused on patients with potentially difficult airway, such as pregnant or obese patients or those with anatomical changes in the maxillofacial and neck regions. Modern applications such as 3D ultrasound may be useful in the complex evaluation of upper and lower airway anatomy with accurate prediction of difficult intubation and correct calculation of tracheal tube size and validation of tube position. Acute airway interventions under real-time ultrasound guidance may become standard procedures in emergency and intensive care settings due to their relative ease of use. Endobronchial ultrasound is becoming widely used by pulmonary physicians. Its place in the diagnostic armamentarium is already firmly stated, and its applications are evolving. Its accuracy and good safety profile facilitate its further spread. Radial probe EBUS is used to navigate to SPNs and to evaluate airway invasion and linear EBUS is a powerful tool for primary staging and restaging of lung cancer and for sampling of all indeterminate mediastinal lesions.

Some of the ultrasound techniques described above are already well established in perioperative practice. The simple reliable evaluations with short learning curve involve ultrasound location of the cricothyroid membrane prior to expected difficult airway management, confirmation of endotracheal tube placement, and perhaps postoperative examination of vocal cords. Other techniques have shown varying reliability and are still more or less at experimental stage. Main limitation of ultrasound examination lies in the fact that it is significantly user-dependent technique and its overall reproducibility is lower than in that of other radiological methods.

## Abbreviations

ACCP: American College of Chest Physicians
BACT: Bougie-assisted cricothyrotomy
BMI: Body mass index
CT: Computed tomography
EBUS: Endobronchial ultrasound
ENB: Electromagnetic navigation bronchoscopy
ICU: Intensive care unit
MRI: Magnetic resonance imaging
PDTS: Percutaneous dilatational tracheostomy
PET/CT: Positron emission tomography/computed tomography
SBRT: Stereotactic body radiation therapy
SPN: Solitary pulmonary nodule
TBB: Transbronchial biopsy
TBNA: Transbronchial needle aspiration
TRUE: Tracheal rapid ultrasound exam
US: Ultrasound.
